# Water-based slurries for high-energy LiFePO4 batteries using embroidered current collectors

**DOI:** 10.1038/s41598-020-62553-3

**Published:** 2020-03-27

**Authors:** Noemí Aguiló-Aguayo, Dominic Hubmann, Fahad Ullah Khan, Stefan Arzbacher, Thomas Bechtold

**Affiliations:** 1 Research Institute of Textile Chemistry and Textile Physics, University of Innsbruck, Hoechsterstrasse 73, 6850 Dornbirn, Austria; 2 illwerke vkw Endowed Professorhip for Energy Efficiency, Research Center Energy, Vorarlberg University of Applied Sciences, Hochschulstrasse 1, 6850 Dornbirn, Austria

**Keywords:** Electrochemistry, Energy

## Abstract

Greater specific energy densities in lithium-ion batteries can be achieved by using three-dimensional (3D) porous current collectors, which allow for greater areal mass loadings of the electroactive material. In this paper, we present the use of embroidered current collectors for the preparation of thick, pouch-type Li-ion batteries. Experiments were performed on LiFePO_4_ (LFP) water-based slurries using styrene-butadiene rubber (SBR) as binder and sodium carboxymethyl cellulose (CMC) as thickener, and formulations of different rheological characteristics were investigated. The electrochemical performance (cyclic voltammetry, rate capability) and morphological characteristics of the LFP half-pouch cells (X-ray micro computed tomography and scanning electron microscopy) were compared between the formulations. An optimum electrode formulation was identified, and a mechanism is proposed to explain differences between the formulations. With the optimum electrode formulation, 350 µm casted electrodes with high mechanical stability were achieved. Electrodes exhibited 4–6 times greater areal mass loadings (4–6 mAh cm^−2^) and 50% greater electroactive material weight than with foils. In tests of half- and full-pouch embroidered cells, a 50% capacity utilization at 1C-rate and 11% at 2C-rate were observed, with a full recovery at C/5-rate. The cycling stability was also maintained over 55 cycles.

## Introduction

Aspects such as the trade-off between energy and power density, cost effectiveness and eco-friendly processing have to be addressed to make lithium-ion batteries practicable. Batteries for electric vehicles require on the one hand specific energies of about 225 Wh kg^−1^ to ensure the ratio between weight and battery autonomy^1^, and on the other hand, the costs should be less than 300€ kwh^−1^, to be more affordable.

The preparation of thick electrodes by increasing mass loadings is one of the strategies to achieve these goals. However, metal foils are universally employed as current collectors in batteries, and they do not permit large electrode thicknesses because of risk of delamination of the electrode material, and the poor electrical conductivity of some electroactive materials^2^. Another strategy is to use three-dimensional (3D) porous structures, which allow for higher mass loadings per unit area. Metal foams have been previously investigated with mass loadings up to 30–50 mg cm^−2^, i.e. five times the areal energy densities of conventional batteries^3^. Nevertheless, the non-uniform porosity in foams makes the coverage of the electrode material inside the structure difficult, and limits the performance of the battery. In addition, after being cut out to the desired geometry, the open ends of the metal foam can damage the separator. 3D electrode configurations employing nanostructured materials, such as 3D microbatteries with silicon nanowires or carbon microrods also permit to increase the energy and power densities up to 100 times greater than conventional batteries^4,5^. However, the wetting or impregnation of the entire architecture with the electrolyte is a challenge. The total capacity is also low, between 10 µAh-10 mAh, which limits their application to miniature electronic devices. The use of conductive energy textiles is another strategy for the preparation of batteries with large mass loadings^6–8^. Hu and coworkers^9^ prepared a Li-ion battery by dip-coating carbon nanotubes on a polyester fabric. The electrodes had a thickness of 600 µm, and exhibited very high mass loadings of about 168 mg cm^−2^. However, the feasibility of large-scale manufacturing is one of the concerns to make these systems commercially viable. Thick electrodes can also be produced via 3D printing techniques, and templating approaches. Wei and coworkers^10^ presented the performance of 3D printed thick electrodes with areal capacities of 4.45 mAh cm^−2^. Demortière and co-workers demonstrated the preparation of thick electrodes with areal capacities of 20 mAh cm^−2^ using a templating method to create 3D structures. Despite the advantages of the mentioned technologies, they also increase the battery costs, and cannot be easily implemented in large-scale battery manufacturing facilities.

We recently proposed the use of 3D embroidered structures as current collectors as another alternative to prepare thick electrodes^11–13^. In contrast to metal foams, there are no open wire ends due to the near-net-shape manufacturing of the embroidered structures. However, electrode formulations with selection criteria different than those for thin electrodes have to be developed to ensure proper layer consistency, uniformity, and coating performance.

Although aqueous processing has been adopted for the manufacturing of anodes made of graphite, most cathodes are still prepared using polyvinylidene fluoride (PVDF) as binder and N-Methyl-2-pyrrolidone (NMP) as the organic solvent. NMP is toxic and expensive. Replacing NMP with water is believed to reduce to half the costs of the electrode processing, it will be more environmentally friendly, and it will allow easier situating of battery manufacturing plants^14^. The use of SBR and CMC mixtures have been positively evaluated to substitute the PVDF binder in thin cathodes^15–17^ and thin graphite anodes^18–21^. However, there are few studies concerning water-based formulations for the preparation of thick electrodes. Guyomard and coworkers^22^ compared the electrochemical performance of thick LiFePO_4_ (LFP) electrodes and rheological properties of water-based formulations prepared with a combination of polyvinyl alcohol and polyethylene glycol (PEG) as binder and two different thickeners. Although they achieved performance similar to non-aqueous slurries, the formulations were not appropriate for practical applications, since they contained PEG, which can be dissolved in some lithium-ion electrolytes.

We present here a systematic investigation on water-based LiFePO_4_ formulations for the preparation of thick pouch-type electrodes using embroidered current collectors. LiFePO_4_ (LFP) due to its safety and long cycle life, is considered the most promising Li-ion technology for large-format batteries^23^. LFP/graphite cells are the only ones that pass all the safety tests without thermal runaway, which is of especial interest in automotive applications^24^. LFP has the additional advantage that it can be processed with water^25^. Formulations using five different mass ratios of SBR:CMC, at fixed LFP and water contents, were investigated. Rheological properties, electrochemical behavior and morphological characteristics for the different formulations are shown. Optimum cathode formulations were identified and an explanation of the mechanism behind is proposed. Full-pouch cells with anode slurries showing the same CMC:SBR mass ratio as optimum cathode formulations were also prepared and electrochemically characterized. Results demonstrate a promising performance of the half and full-pouch thick cells, showing a successful approach to prepare large-format high specific energy batteries with a more eco-friendly and cost-effective electrode processing.

## Materials and Methods

### Slurry and electrode preparation

Five different water-based slurry formulations with 35 wt.% solid content and 65 wt.% water content were investigated with variations in the proportions of styrene-butadiene-rubber (SBR) and sodium carboxymethyl cellulose (CMC) (Table 1). LFP particles (Pholicat FE100, beLife, Belgium) were used as obtained. The LFP particles were carbon-coated, with an average diameter (D50 with ultrasounds) of 2.76 µm, a carbon percentage of 2.14%, and a BET specific surface area of 20.9 m^2^ g^−1^, (values provided by the supplier). The carbon additive used was C65 (Super C65, Timcal, Switzerland) with a BET specific surface area of 62 m^2^ g^−1^. The SBR binder was obtained as an emulsion with 48 wt.% solids (MTI Corporation, USA). The sodium CMC used as thickener had a degree of substitution (DS) 0.6–0.9, and a viscosity of 1500–3000 mPas at a concentration of 1 wt.% and at 25 °C (Sigma-Aldrich Chemie GmbH, Steinheim, Germany). The CMC solution was prepared with sodium CMC soaked overnight in deionized water at room temperature at the desired concentration. The slurries were obtained by first mixing LFP with C65, and then adding the desired weight concentration of CMC and SBR afterwards. The slurries were finally mixed in an ultra high shear mixer at 10000 rpm for 5 min (S25N-25G Ultra-turrax, IKA-Werke GmbH & Co.KG, Germany).

**Table 1:** Water-based electrode formulations used in the experiments.

SBR:CMC mass ratio	Wet slurry composition^a^		Dried electrode composition^b^	
	SBR/wt.%	CMC/wt.%	SBR/wt.%	CMC/wt.%
(A) 1: 0	1.75	0	5	0
(B) 4: 1	1.4	0.35	4	1
(C) 1: 1	0.875	0.875	2.5	2.5
(D) 1: 4	0.35	1.4	1	4
(E) 0: 4	0	1.75	0	5

^a^All wet slurries contained the following: LFP (30.1 wt.%), C65 (3.15 wt.%), SBR + CMC (1.75 wt.%), water (65 wt.%).

^b^All dried electrodes contained the following: LFP (86 wt.%), C65 (9 wt.%), SBR + CMC (5 wt.%).

**Figure 1:**
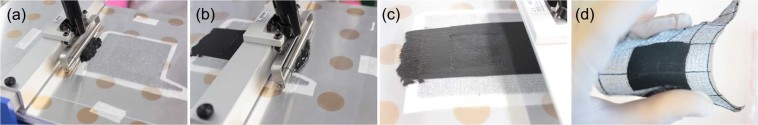
(**a–c**) Application of the LFP slurry SBR:CMC 4:1 (w:w) on the embroidered current collectors using a film applicator. A plastic sheet was used as template to define a desired electrode area of 4 × 4 cm^2^. (**d**) Final electrode after drying and pressing.

The embroidered current collectors were prepared by embroidery (Texible GmbH, Hohenems, Austria). Two layers of aluminium wires (80 µm in diameter) were embroidered on a polyester fabric. The Al wires form a mesh as shown in Fig. 1. The distance between Al wires was about 0.4 mm. The original thickness of the embroidered structure alone was 870 µm. Thicknesses were obtained with a thickness gauge under a normal pressure of 17.5 g cm^−2^ (Karl Schröder KG Weinheim, Germany).

The slurry was applied on an area of about 4 × 4 cm^2^ of the embroidered current collectors using a film applicator (Coatmaster 510, Erichsen GmbH & Co.KG, Hemer, Germany) with a spiral film applicator Model 358, at a speed of 10 mm/s. Figure 1 shows an example of the application process applied to the embroidered current collectors. A plastic sheet was used as a template to define the application area of 4 × 4 cm^2^. The electrodes were first pre-dried at 80 °C and then pressed at 13.3 MPa for 3 min. The electrodes were dried in an argon vacuum oven for 4 hours at 150 °C, and finally pressed at 25 MPa for 3 min at room temperature. The electrodes were pressed in a laboratory press Polystat 200 T (Sevitec Maschinenservice GmbH, Germany). The final electrode composition after drying is shown in Table 1 for each slurry formulation. After the final drying and pressing, the electrodes had a total thickness of about 350–400 µm. All electrodes exhibited similar mass loadings and total capacities.

Anodes for the full pouch cell were prepared with copper embroidered current collectors using the same design as the aluminum current collectors. The anode slurry formulation used was 32.5 wt.% graphite (E-SLS30 Graphite, Timcal, Switzerland), 0.35 wt.% conductive carbon (Super C65, Timcal, Switzerland), 0.35 wt.% sodium CMC, 1.39 wt.% SBR (48 wt.% aqueous emulsion, MTI Corporation, USA), 16.4 wt.% deionized water. All materials were used as received. The SBR:CMC mass ratio used was 4:1. Anodes were dried in an argon oven vacuum for 4 hours at 150 °C and pressed at 4 MPa for 3 min at room temperature.

### Rheological measurements

The rheological measurements were carried out at room temperature using a rheometer (MCR 302, Anton Paar GmbH, Germany) with the 25 mm diameter parallel plate measuring system. The gap between plates was fixed at 1 mm for all measurements. The viscosity and shear stress versus the shear rate were obtained with a rotational test. The storage and loss modulus (*G’, G”*) versus the shear strain were measured under amplitude sweeps at a constant angular frequency of 10 rad/s. The *G’* and *G”* as a function of the angular frequency were acquired at a constant shear strain of 1% of the gap distance to ensure the linear viscoelastic (LVE) regime.

### Morphological characterization

Scanning electron microscopy (SEM) images were performed using JEOL JSM-7100F microscope operated at 20 keV. Laser scanning microscope (LSM) images LSM (VK-X100 series LSM 3D Profile Measurement from KEYENCE) was employed for optical observations. X-ray micro computed tomography (µCT) scans were performed using GE’s phoenix nanotom m (now distributed by BarkerHughes, a GE Company) at a focal spot size of 1.7 µm. All scans were done at a tube voltage of 80 kV and 110 µA tube current. The detector of the manotom m (GE’s DXR500L) features 3070 × 2400 pixel, 100 µm pixel pitch size, and a dynamic range of 10.000:1. Full rotation scans with 1600 projections, an average of 3 images per angular increment and an exposure time of 1000 ms per image yielded a total scan duration of 1 h 40 min. The cone beam geometry resulted in a geometrical magnification factor of 66.7 and a voxel size of 1.5 × 1.5 × 1.5 µm^3^. Volume reconstruction was done with the commercially available phoenix datos|x software package (BakerHughes, a GE Company). For the reduction of image noise, the 3D image data was post processed using a median filter with a kernel size of 5 voxels.

### Pouch cell preparation and electrochemical characterization

The pouch cells were assembled in an argon glove box (MBraun Unilab Plus, M. Braun Inertgas-Systeme GmbH, Germany) with moisture and oxygen levels lowers than 0.1 ppm. Li foil (Sigma-Aldrich Chemie GmbH, Steinheim, Germany) with dimensions of 4.5 cm width, 4.5 cm length, and 0.6 mm thickness was used as anode and reference electrode for the half-cells experiments. For full-cell experiments, anodes were prepared with graphite electroactive powders as described in the previous section. Celgard 2400 (samples courtesy of Celgard LLC, France) was used as a separator. The electrolyte was 1 M LiPF_6_ in 1:1 v/v ethylene carbonate and diethyl carbonate EC/DEC (Sigma-Aldrich Chemie GmbH, Steinheim, Germany). The same amount of electrolyte (about 8 mL) was used in all the cells. Cyclic voltammograms (CVs), and galvanostatic charge and discharge cycles were performed on a VSP Bio-Logic system with a EC-Lab software (Gamec Analysentechnik, Germany). The C-rate was calculated considering the theoretical specific capacity of LiFePO_4_ (170 mAhg^−1^).

## Results and Discussion

### Embroidered current collector characterization

**Figure 2:**
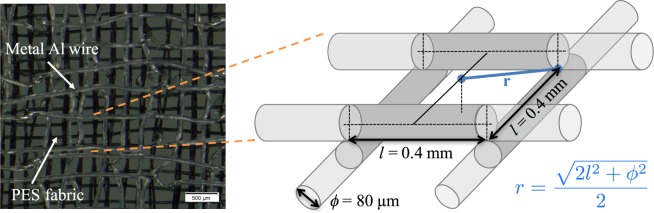
Micrograph of the embroidered current collector and schematic drawing (not scaled) of the structure showing the diameter of the Al wire (*ϕ*), distance between wires (*l*), and the maximum distance between the electrode material and current collector (*r*).

Figure 2 shows the embroidered structure used as current collector. The capacity of the battery depends on the volume available for accommodation of electrode material within the 3D embroidered structure. The segment *r* in Fig. 2 illustrates the farthest distance between the electrode material and the current collector. For the present embroidered layout with a wire diameter of 80 µm, *r* was about 0.286 mm and the distance between wires was 0.4 mm. In planar geometries, the *r* value corresponds to the thickness of the electrode material. The distance *r* in the embroidered structures is the distance between two points, whereas in metal foils the electrode thickness is the separation between two planes. Therefore, in case of metal foils, more of the electrode material is subjected to the farthest distance. For the present layout, mass loadings between 25–35 mg cm^−2^ were feasible. These mass loadings correspond to theoretical areal capacities of 4–6 mAh cm^−2^, which is 4–6 times larger than that reported for conventional planar electrodes^20^. The mass of the current collector alone was 12.3 ± 0.5 mg cm^−2^, and the proportion of the electroactive material weight was 60–70% of the total electrode mass. In comparison, the proportion of the electroactive material in conventional electrodes is in the range of 40%^26^.

The mechanical stability of the electrode material in thick batteries depends on the surface area available for contact with the current collector. For metal foils, the surface area is given by the geometric area i.e. the product of the length and width. In embroidered structure, the surface area of their elements (metal wires and filaments of the background fabric) may be calculated by multiplying the cylindrical surface area of the wire/filament (π*ϕl*/2) with the number of wires/filaments (*N*). For the same geometric dimensions (length and width), the metal surface area in the present embroidery layout was 1.26 times larger than that in a metal foil. The polyester (PES) background fabric used for embroidery may also contribute to enhance the stability with a 4.28 times larger surface area than the metal foil. Because of the larger surface area available, the embroidered electrodes, despite their thickness, are likely to maintain their integrity even if bent.

### Rheological properties

**Figure 3:**
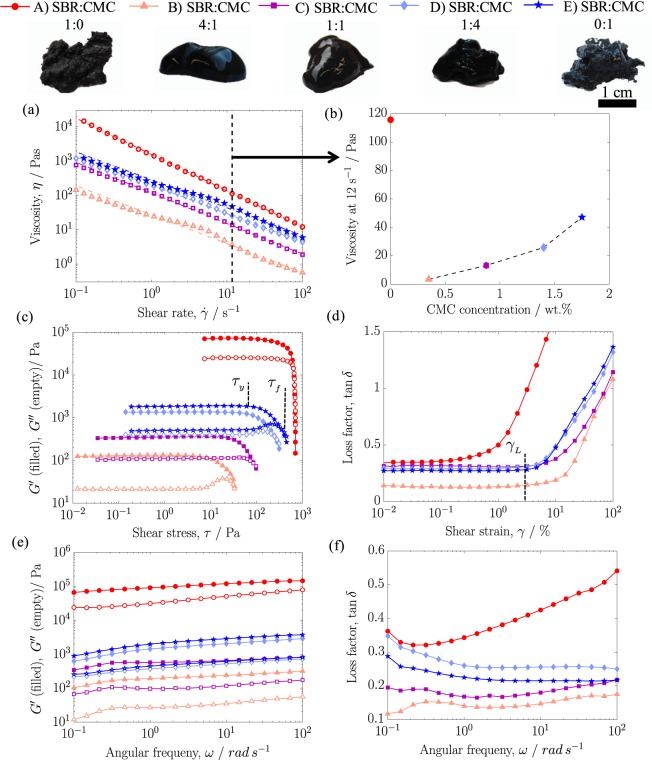
(Top) Images of the slurries for each formulation. (**a**) Viscosity versus shear rate, (**b**) viscosity at 12 s^−1^ versus sodium CMC concentration, (**c**) *G’* and *G”* with increasing shear stress, (**d**) loss factor with increasing shear strain, (**e**) *G’* and *G”* and (**f**) loss factor with increasing angular frequency at 1% shear strain.

The slurry formulation must exhibit a proper flowability to ensure the good permeation through the embroidered structure, and at the same time there should be negligible particle settling and the proper consistency to maintain the desired thickness. These factors may be evaluated from the rheological properties displayed in Fig. 3 of the formulations. The viscosity of the slurries versus shear rate (0.1 to 100 s^−1^) is shown in Fig. 3a. From this range the shear rate of 12 s^−1^ was chosen for further analysis (Fig. 3b), since it corresponded to the rate of shear in our application method. That was calculated by dividing the application speed (10 mm s^−1^) by the original thickness of the current collectors (850 µm). In Fig. 3b it may be observed that viscosity of Formulation A (with no CMC) was far greater than that of the other formulations. Water was observed to be displaced from the slurry in the course of measurements on Formulation A, which was not observed with the other formulations. Thus, the significantly greater viscosity of Formulation A was due to higher particle concentrations as the water was removed. In essence, Formulation A rather behaved like a sand/water mixture rather than a solid-gel structure. Therefore, we observed that CMC was required to provide a solid-gel structure to the slurry. Adding a small amount of CMC (i.e. SBR:CMC 4:1) was already enough to significantly decrease the slurry viscosity, and form a network structure. Viscosities of Formulations B and C, which were between 5–15 Pas at a shear rate of 12 s^−1^, were seen to exhibit the best consistency in the casting trials. These viscosities are about 100 times greater than what is used in tape-casting of thin electrodes^16,27^, and about 10 times greater than in the tape-casting of thick electrodes on metal foils^22^. Therefore, viscosity values different than the ones used for planar current collectors were required for the preparation of thick electrodes using embroidered current collectors.

Figure 3c shows storage (*G’*) and loss (*G”*) moduli versus the shear stress for the different formulations. From these measurements, the yield point (*τ*_*y*_), which indicates the end of the linear viscoelastic (LVE) region, and the flow point (*τ*_*f*_), which is the point where *G’* and *G”* crossover, can be determined. From those points, the flow transition index, which is the ratio *τ*_*y*_*/τ*_*f*_, was calculated. The flow transition index provides a measure of the brittle fracturing behavior of a slurry. The closer the values are to 1, the more abrupt is the drop observed for *G’* and *G”* after the LVE region. This is indicative of a greater tendency of a slurry for brittle fracture, and its low spreadability. The flow transition index of formulation A was close to 1, while all other formulations displayed values between 0.11–0.17. Therefore, except for formulation A that showed a brittle fracturing behaviour, all formulations exhibited similar spreadabilities, though, at different shear stresses. The larger the CMC content, the higher the flow point τ_f_, and therefore the greater the squeezing forces required for spreading the slurry. That was related to the thickening action of the CMC on the water phase, in agreement with the viscosity values.

Figure 3d shows the loss factor, tan δ = *G”/G’*, as a function of the shear strain. The limiting shear strain value (*γ*_*L*_) indicates the limit of the LVE region. Formulation B exhibited the lowest *G”*, but also the lowest loss factor (i.e. larger *G’* in comparison to *G”*). That is indicative of greater structural strength in formulation B, and points towards a greater propensity to flow with a more consistent three-dimensional network structure. Thus, a better filling of the uneven parts of the current collector is expected with this formulation. Figure 3e,f display *G’* and *G”* and the loss factor (tan δ = *G”/G*) as a function of the angular frequency. Formulation B exhibited the smallest values at the lowest frequencies, which is indicative of better particle dispersion stability.

From the rheological studies, we could derive that although both formulations B and C provided optimum viscosities for casting on embroidered current collectors; formulation B exhibited additional rheological features (spreadability at lower shear stress, better flowability with a more consistent network structure, and less likely to particle sedimentation) to be selected as the optimal formulation. To confirm the rheological findings, we performed further morphological and electrochemical characterization of the electrodes.

### Morphological properties

**Figure 4:**
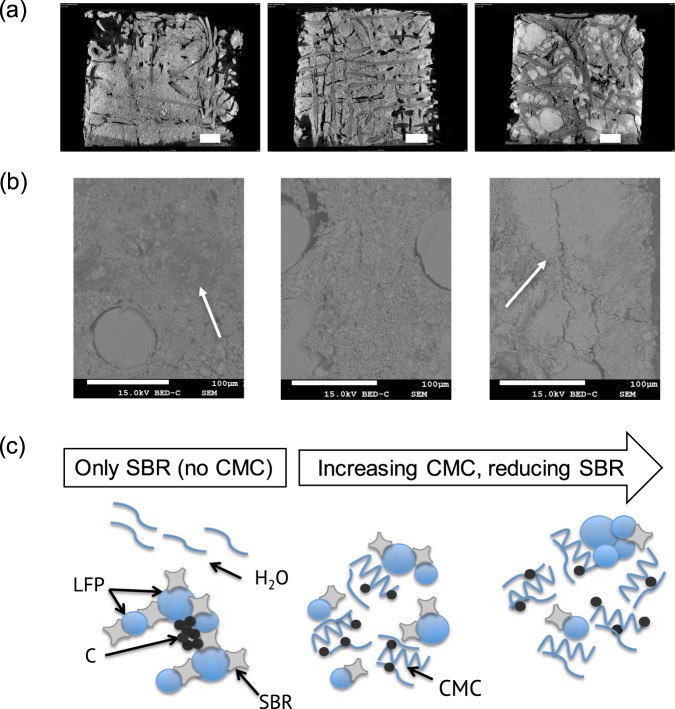
(**a**) From left to right, µCT front images of the dried electrodes corresponding to formulations A, B, C. Bar scale: 450 µm. (**b**) From left to right, SEM images obtained by backscattered electrons (BE) of the cross-section of dried electrodes corresponding to formulations A, B, D. (**c**) Mechanism for the explanation of the particle agglomeration depending on the CMC and SBR content.

Figure 4 shows (a) µCT and (b) SEM images of dried electrodes obtained using different formulations. Formulations C, D and E exhibited similar features. Additional images are provided in Supplementary Information (Fig. S1). Depending on the CMC concentration, three different features were identified in the dried electrodes: (i) when CMC was not present (i.e. formulation A) higher agglomerations of the conductive carbon were observed, (ii) when SBR:CMC was 4:1 (i.e. formulation B) a more uniform distribution of the particulates (both LFP and carbon) was achieved, and (iii) when CMC was 2.5 wt.% and larger and SBR was 2.5 wt.% and lower (i.e. formulations C, D and E) higher LFP agglomerations and cracks were observed. Since the concentration of LFP and conductive carbon was kept constant in all formulations, differences observed in the images resulted from differences in the particle distributions within the electrode. Due to LFP agglomerations observed in the wet slurry as shown in LSM images provided in Supplementary Information (Fig. S2), it is expected that agglomerations were already present and not generated during electrode drying.

The proposed mechanism for the particle agglomeration is shown in Fig. 4c. SBR has a tendency to be attached on particulates. Without CMC (i.e. formulation A) a rapid mechanical separation between particulate matter and liquid phase (already observed in the rheological studies) occurred, which led to a non-uniform distribution of the conductive carbon within the electrode. The dispersing agent used in SBR solutions may also have a role in the LFP agglomeration propensity. When SBR concentrations were larger than 4 wt.% (i.e. Formulation A, B), no LFP agglomeration was observed.

On addition of CMC to the formulations, the water phase thickens (observed in rheological studies), and thus the mechanical separation between water and solids was prevented and particles got dispersed. Previous studies stated that CMC adsorbs more preferably on carbon particles than SBR^21^. This may contribute to ease the distribution of carbon particles within the electrode when CMC is present. However, an excess of CMC hindered the mechanical separation of LFP particulates during high-shear stirring (lower flowability with higher CMC content), and LFP agglomerations occurred.

### Electrochemical performance

**Figure 5:**
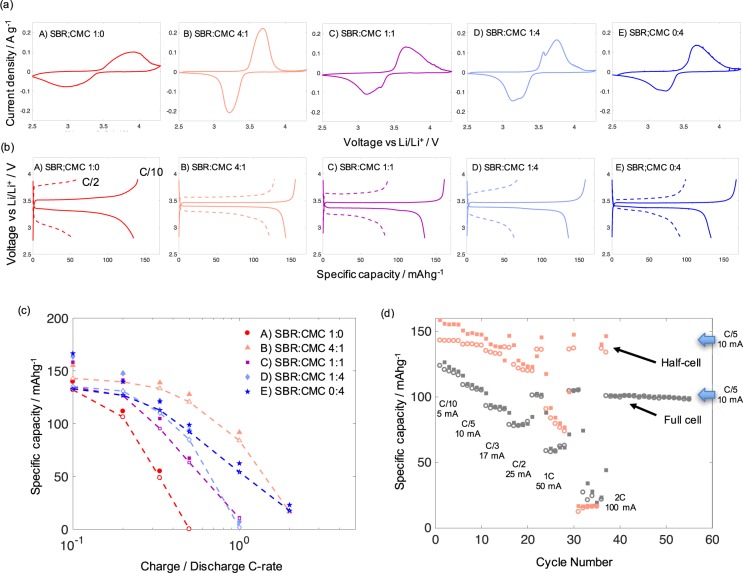
(**a**) Cyclic voltammograms (2^nd^ cycle) at a scan rate of 0.1 mVs^−1^, (**b**) galvanostatic charge and discharge cycles at C/10 and C/2-rate, (**c**) capacity utilization versus C-rate during charge (close symbols) and discharge (open symbols) for half-cells prepared with electrodes using formulations from Table 1. (**d**) Cycle stability at different C-rates for electrodes prepared with SBR:CMC 4:1 mass ratio for a half- and full -cell.

Figure 5 shows the electrochemical performance of electrodes using Formulations A to E. Table 2 summarizes the electrochemical properties from CV measurements and charge/discharge cycles (Fig. 5a,b). Maximum values of the specific capacity were about 150 mAh g^−1^ at the lowest C-rate (C/5-rate). This value is close to the theoretical specific capacity of LFP. Therefore, we can consider that we reached 100% electrode capacity utilization at that C-rate. Better specific capacities at this C-rate could be achieved by using smaller LFP particles as raw material.

**Table 2:** Electrochemical properties from CVs and charge/discharge cycles.

Formulation	A	B	C	D	E
Cathodic/anodic peak potential/V	3.91/2.96	3.68/3.22	3.67/3.12*	3.75*/3.19*	3.69/3.25*
Peak potential separation/V	0.95	0.46	0.55	0.56	0.44
Cathodic/anodic peak current densities/Ag^−1^	0.100/ −0.077	0.221/−0.208	0.132/−0.109	0.165/−0.146	0.134/−0.098
Specific charge/discharge capacity at C/10 and C/5 rates/mAhg^−1^	140.4/134.7	155.9/143.0	161.5/135.4	158.2/135.5	166.5/132.9
Coulombic efficiency (CE)	CE: 95.9%	CE: 91.7%	CE: 83.4%	CE: 85.7%	CE: 79.8%
	60.3/54.5	127.8/121.9	86.7/83.9	67.3/63.3	98.8/92.3
	CE: 90.4%	CE: 95.3%	CE: 96.8%	CE: 94%	CE: 93.4%
Charge/discharge middle point voltage at C/10 and C/5 rates/V	3.54/3.30	3.46/3.39	3.46/3.36	3.47/3.37	3.46/3.37
Voltage efficiency (VE)	VE: 93.2%	VE: 98.0%	VE: 97.1%	VE: 97.1%	VE: 97.3%
	3.82/3.00	3.57/3.27	3.64/3.21	3.71/3.14	3.54/3.29
	VE: 78.5%	VE: 91.6%	VE: 88.2%	VE: 84.6%	VE: 92.9%

*The value corresponds to the highest peak.

Formulation B exhibited the best electrochemical performance: the most pronounced redox peaks, largest specific charge/discharge capacities, and best efficiencies. The gravimetric energy density for this formulation achieved at C/5-rate was 314 Wh kg^−1^ (calculated with the specific discharge capacity of 142.9 mAh g^−1^, a discharge middle voltage at C/5-rate of 3.39 V, and 65% of electroactive material with respect to the total electrode weight). This corresponds to more than 1.5 times greater gravimetric energy densities than the ones obtained with conventional LFP planar electrodes (90–160 Wh kg^−1^)^26^. In contrast, the volumetric energy density was about 248 Wh L^−1^ (calculated considering a total electrode thickness of 360 µm), which was two times lower than values obtained with conventional planar electrodes of 50 µm total thickness. However, for applications such as electric vehicles, the gravimetric energy density is more important than the volumetric one. The good performance of this formulation was attributed to the uniform distribution of both carbon and LFP particles in the cathodes, and the small LFP sizes, which facilitated the Li-ion diffusion and decreased the charge-transfer resistance.

Formulation A showed the largest peak separation and overpotentionals (middle point voltages) during cycling. This was due to the poor dispersion of the conductive carbon in the cathodes, which negatively affected their electronic conductivity. Formulations C to E exhibited double peaks during oxidation or/and reduction in the CVs. This was a consequence of the presence of the large LFP agglomerates, which may contribute to provide a bimodal size distribution with primary and secondary LFP particle sizes^28^. Formulations B to E delivered maximum charge capacities at the lowest C-rate (C/10 rate) with low overpotentials. This was attributed to the positive contribution of the CMC to the dispersion of carbon in the cathodes. However, Formulations C to E exhibited a huge loss in capacity, and thus low CE, during the discharge at C/10 rate. This may be consequence of the break-up of the large LFP agglomerates during charge, which may cause a loss of contact with the current collector and conductive carbon.

The dependence of the specific charge/discharge capacities with the C-rate is shown in Fig. 5d. By increasing the C-rate, the specific capacity decreased as expected. However, Formulation B exhibited the smoothest capacity drop when C-rate increased, since the cathodes could sustain the highest current densities as observed in the CVs. The charge/discharge cycles at the different C-rates and the electrochemical impedance spectroscopy measurement are provided in Supplementary Information (Figs. S3 and S4). The rate capability during cycling for Formulation B is shown in Fig. 5d. Current densities up to 0.34 Ag^−1^ (2C-rate) could be applied without degradation of the cells and with a complete capacity recovery at C/5-rate. We have to notice that batteries were submitted at high stress, since they were both charged and discharged at the same high C-rate. A 50% and 11% capacity utilization was reached at 1 C and 2C-rate. The rate capability of full-cells prepared with cathodes and anodes with SBR:CMC 4:1 mass ratio is also depicted in Fig. 5d. A picture of a full-cell is shown in Supplementary Information (Fig. S5). Although the discharge capacities for the full-cell were 11% reduced (capacities were 109 mAhg^−1^ and 107 mAhg^−1^ at C/5-rate for charge and discharge, respectively), a similar rate capability response was obtained. In addition, coulombic efficiencies (98% at C/5 rate) were higher in comparison to half-cells and the cycle stability was maintained over 55 cycles.

## Conclusions

A systematic study on water-based LFP formulations was performed to prepare thick electrodes using embroidered current collectors. Embroidered structures exhibit higher areal surface area than metal foils, allowing for 4–6 times greater areal mass loadings than planar metal foils, with an electroactive material weight of 60–70% with respect to the total electrode weight (50% greater than metal foils).

Five different formulations were investigated using different SBR:CMC mass ratio, while maintaining the same LFP, carbon and water content. Rheology, morphological and electrochemical properties were in agreement. Formulation B (with SBR:CMC 4:1 mass ratio) exhibited the most appropriate characteristics. Concerning rheological properties, formulation B showed the most suitable consistency, spreadability, flowability, network formation and particle sedimentation. Electrodes prepared with formulation B also showed the most uniform particle (LFP and carbon) distribution and best electrochemical performance, with greater specific charge/discharge capacities and rate capabilities. A similar performance was also observed with full-cells using anodes and cathodes prepared with SBR:CMC 4:1 mass ratio, which exhibited a cycle stability over 55 cycles. The results exhibit the best ever reported electrochemical performance of thick LFP electrodes of 350–400 µm total thickness prepared with water-based formulations. Optimized LFP electrodes exhibit a gravimetric energy density of about 314 Wh kg^−1^ at C/5-rate, more than 1.5 times greater than the reported values for conventional LFP planar electrodes. The optimization of the SBR:CMC mass ratio, which defines the optimized rheological properties of the slurry to be applicable to the embroidered current collectors, can be employed with other chemistries as shown in our work in the preparation of the anodes and their performance test in full-cells. From this point further optimizations can be performed, for instance by modifying the initial raw materials used in the formulations (e.g. using smaller LFP particles, or different types of binders, thickeners and conductive additives). The results open new strategies in the development of high-performance thick electrodes with a more-ecofriendly and cost-effective electrode processing.

## Supplementary information

Supplementary Information.
