# Supplementary material for: Water-based slurries for high-energy LiFePO4 batteries using embroidered current collectors
Source: Sci Rep. 2020 Mar 27;10:5565. doi: 10.1038/s41598-020-62553-3 (PMC7101393; doi:10.1038/s41598-020-62553-3)
Supplement: Supplementary file 1 — Supplementary Information. [file 41598_2020_62553_MOESM1_ESM.docx]

**Water-based slurries for high-energy LiFePO4 batteries using embroidered current collectors**

Noemí Aguiló-Aguayo*^a^, Dominic Hubmann^a^, Fahad Ullah Khan^a^, Stefan Arzbacher^b^ and Thomas Bechtold^a^

^a^Research Institute of Textile Chemistry and Textile Physics, University of Innsbruck, Hoechsterstrasse 73, 6850 Dornbirn, Austria

^b^illwerke vkw Endowed Professorhip for Energy Efficiency, Research Center Energy, Vorarlberg University of Applied Sciences, Hochschulstrasse 1, 6850 Dornbirn, Austria.

*corresponding author: [noemi.aguilo-aguayo@uibk.ac.at](mailto:noemi.aguilo-aguayo@uibk.ac.at), [textilchemie@uibk.ac.at](mailto:textilchemie@uibk.ac.at), Tel. +4351250755002

**Supplementary Information**

1. µCT front images of dried cathodes prepared with Formulation D (left) and Formulation E (right)


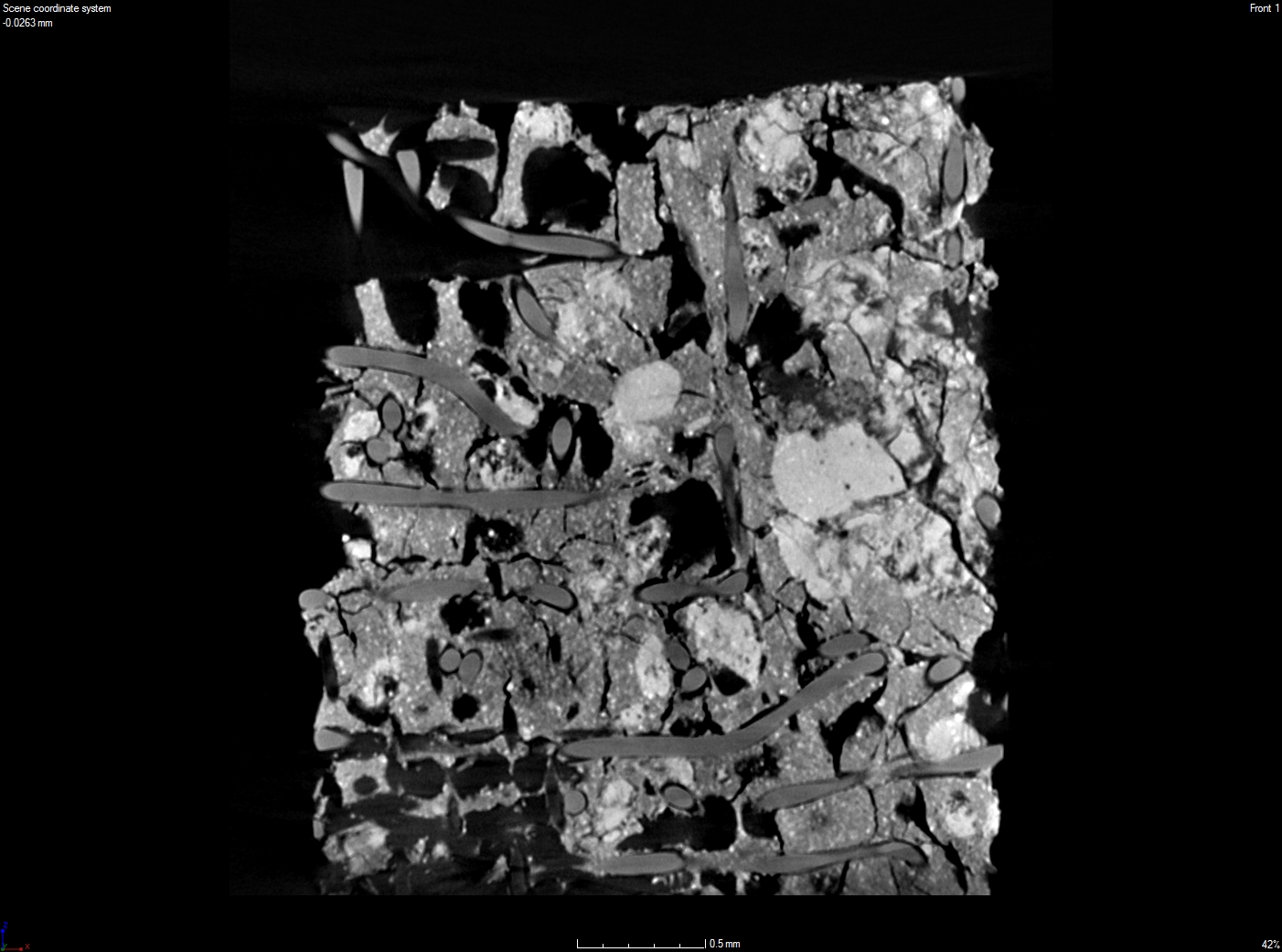


500 µm


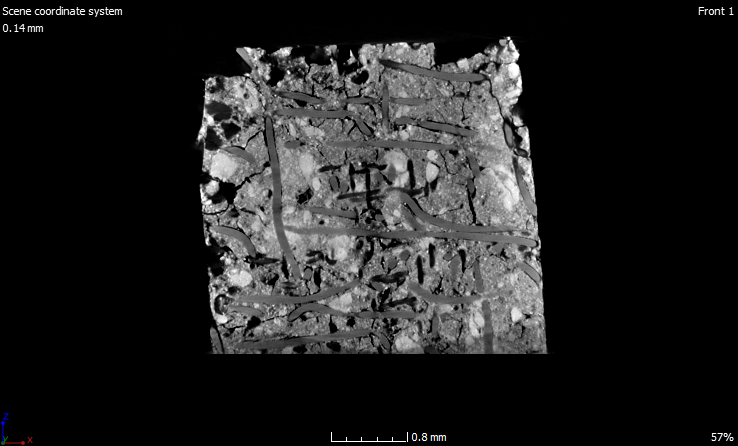


800 µm

1. Cross-section SEM images of dried cathodes prepared with Formulation C (left) and Formulation E (right)

**
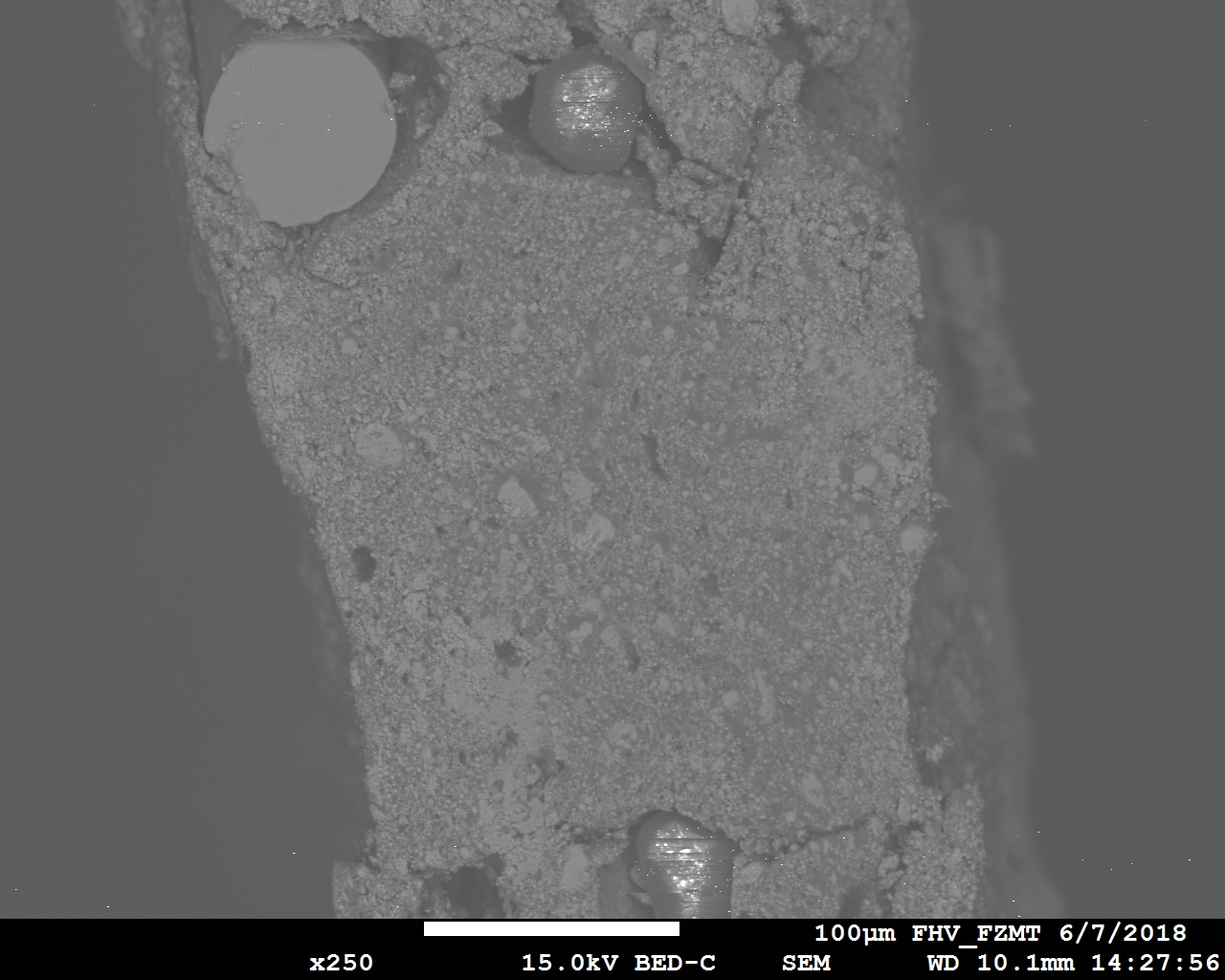

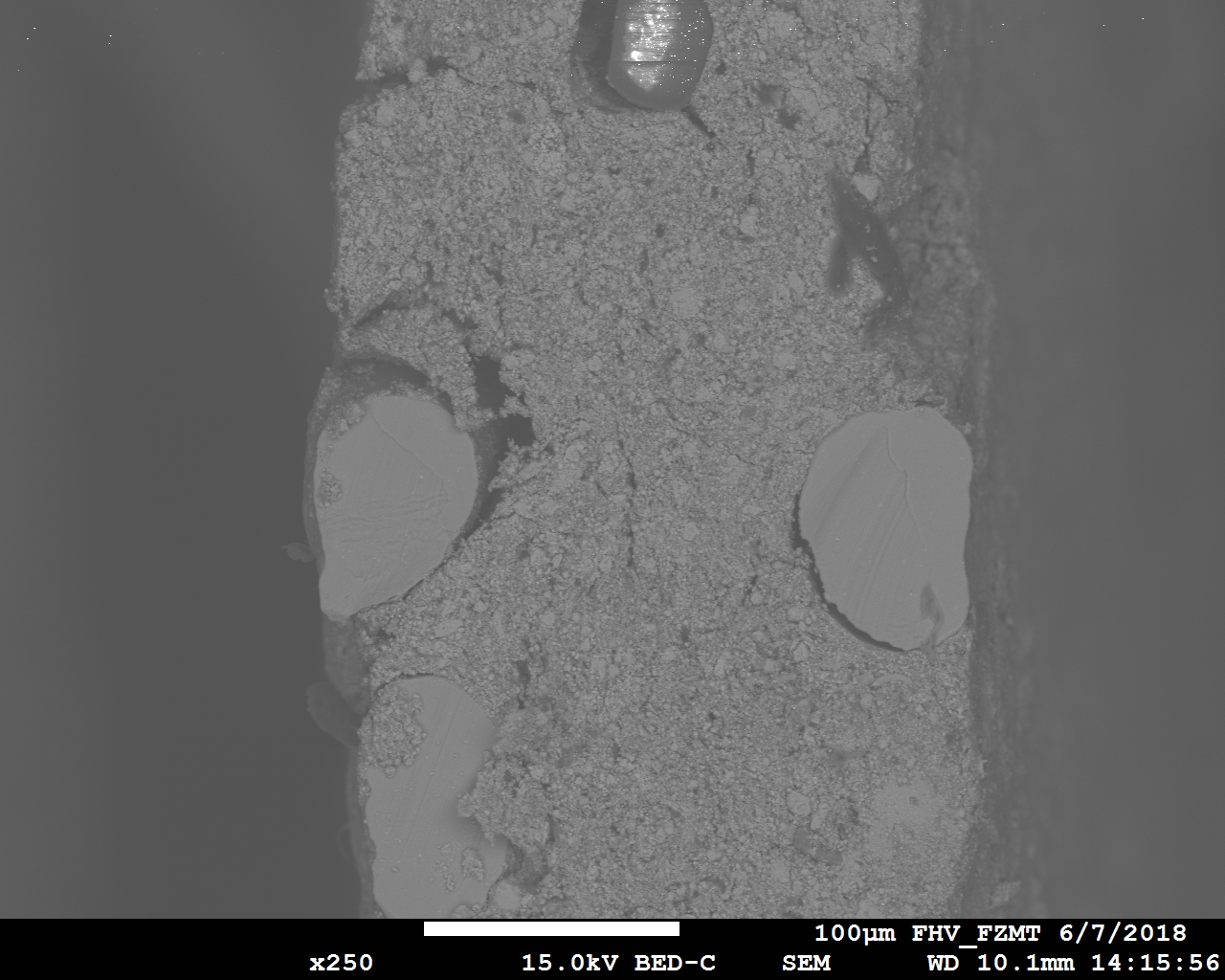
**

**Figure S1.** Additional µCT images and SEM for Formulations C, D and E.

**Figure S2.** 3D LSM images of the wet slurry for Formulation B (left) and Formulation C (right). Slurries from Formulation B exhibited a smoother surface.

**
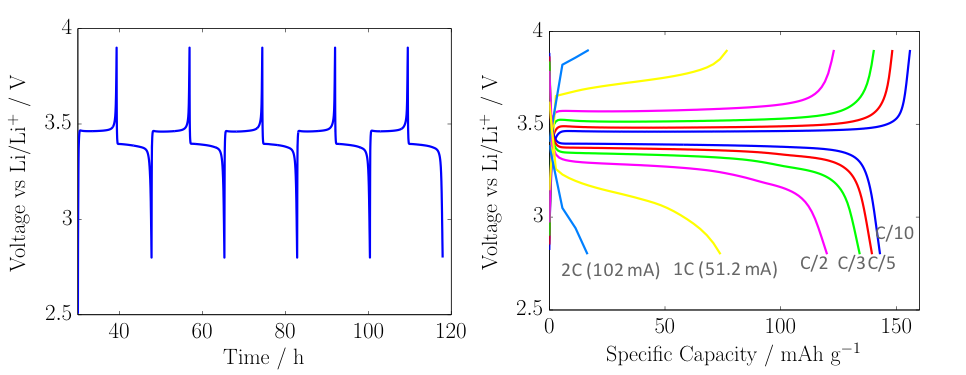
**

**Figure S3.** Charge/discharge cycles at C/5-rate (left) and with increasing C-rate (right) of half-cells prepared with Formulation B.

**
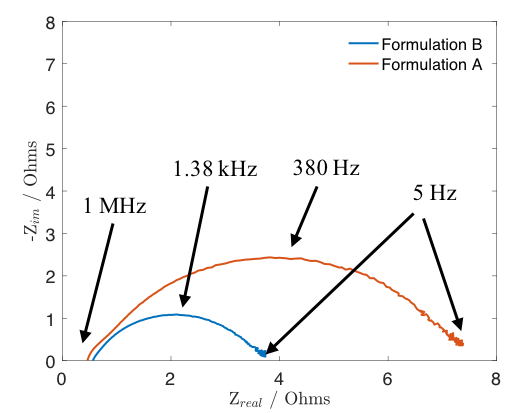
**

**Figure S4.** Electrochemical impedance spectroscopy (EIS) measurements of electrodes from Formulation B and A after charging at 62% SOC at frequencies from 5 Hz to 1MHz. Smaller impedance values are obtained with Formulation B in agreement with charge/discharge profiles and overpotential values.

**Figure S5**. Picture of a full-cell prepared with an anode with a Cu embroidered current collector and graphite electroactive material (left), and a cathode with an Al embroidered current collector and LFP as electroactive material (right).
